# Supplementary material for: Self-Reported Depression among Chinese Women with Recurrent Pregnancy Loss: Focusing on Associated Risk Factors
Source: J Clin Med. 2022 Dec 16;11(24):7474. doi: 10.3390/jcm11247474 (PMC9785000; doi:10.3390/jcm11247474)
Supplement: Supplementary file 1 [file jcm-11-07474-s001.zip › jcm-2073358-supplementary.pdf]

Supplementary materials

1. Questionnaire for patients with recurrent pregnancy loss
2. Questionnaire for healthy women

Questionnaire 1

(For patients with recurrent pregnancy loss)

Part 1

Your Name:

Tel:

Age: □ ≤ 35 years □ ≥ 36 years

Times of Spontaneous Miscarriage: □ 2 times □ ≥ 3 times

History of Stillbirth: □ yes □ no

History of Induced Abortion: □ yes □ no

History of Live Birth: □ yes □ no

Gestational status: □ non-pregnancy □ first trimester □ second or third trimester

Body mass index (BMI): □ < 18.5 kg/m^2 □ 18.5–24 kg/m^2 □ > 24 kg/m^2

Working Hours per day: □ ≤ 8 hours □ > 8 hours

Education Background: □ university and higher □ lower levels of education

Smoking: □ yes □ no

Alcohol Consumption: □ yes □ no

Residence: □ rural □ urban

Household Income: □ ≤ 10000 yuan/month □ > 10000 yuan/month

Part 2

Instructions: For each item below, please place a check mark (√) in the column which best describes how often you felt or behaved this way during the past several days.

**Place check mark (√) in correct column**	**Never or rarely**	**Sometimes**	**Often**	**Most of the time**
1. I feel down-hearted and blue.				
2. Morning is when I feel the best.				
3. I have crying spells or feel like it.				
4. I have trouble sleeping at night.				
5. I eat as much as I used to.				
6. I still enjoy sex.				
7. I notice that I am losing weight.				
8. I have trouble with constipation.				
9. My heart beats faster than usual.				
10. I get tired for no reason.				

11. My mind is as clear as it used to be.				
12. I find it easy to do the things I used to.				
13. I am restless and can't keep still.				
14. I feel hopeful about the future.				
15. I am more irritable than usual.				
16. I find it easy to make decisions.				
17. I feel that I am useful and needed.				
18. My life is pretty full.				
19. I feel that others would be better off if I were dead.				
20. I still enjoy the things I used to do.				

Note: In scoring the SDS, a value of 1, 2, 3 and 4 is assigned to a response depending upon whether the item is worded positively or negatively.

For items 1, 3, 4, 7, 8, 9, 10, 13, 15, 19 the scoring is:

- Never or rarely =1
- Sometimes =2
- Often = 3
- Most of the time =4

For items 2, 5, 6, 11, 12, 14, 16, 17, 18, 20 are reverse scored as follows:

- Never or rarely =1
- Sometimes = 2
- Often =3
- Most of the time =4

Questionnaire 2

(For healthy women)

Part 1

Your Name:

Tel:

Age: □ ≤ 35 years □ ≥ 36 years

Gestational status: □ non-pregnancy □ first trimester □ second or third trimester

Body mass index (BMI): □ < 18.5 kg/m^2 □ 18.5–24 kg/m^2 □ > 24 kg/m^2

Working Hours per day: □ ≤ 8 hours □ > 8 hours

Education Background: □ university and higher □ lower levels of education

Smoking: □ yes □ no

Alcohol Consumption: □ yes □ no

Residence: □ rural □ urban

Household Income: □ ≤ 10000 yuan/month □ > 10000 yuan/month

Part 2

Instructions: For each item below, please place a check mark (√) in the column which best describes how often you felt or behaved this way during the past several days.

**Place check mark (√) in correct column**	**Never or rarely**	**Sometimes**	**Often**	**Most of the time**
1. I feel down-hearted and blue.				
2. Morning is when I feel the best.				
3. I have crying spells or feel like it.				
4. I have trouble sleeping at night.				
5. I eat as much as I used to.				
6. I still enjoy sex.				
7. I notice that I am losing weight.				
8. I have trouble with constipation.				
9. My heart beats faster than usual.				
10. I get tired for no reason.				
11. My mind is as clear as it used to be.				
12. I find it easy to do the things I used to.				

13. I am restless and can't keep still.				
14. I feel hopeful about the future.				
15. I am more irritable than usual.				
16. I find it easy to make decisions.				
17. I feel that I am useful and needed.				
18. My life is pretty full.				
19. I feel that others would be better off if I were dead.				
20. I still enjoy the things I used to do.				

Note: In scoring the SDS, a value of 1, 2, 3 and 4 is assigned to a response depending upon whether the item is worded positively or negatively.

For items 1, 3, 4, 7, 8, 9, 10, 13, 15, 19 the scoring is:

- Never or rarely =1
- Sometimes =2
- Often = 3
- Most of the time =4

For items 2, 5, 6, 11, 12, 14, 16, 17, 18, 20 are reverse scored as follows:

- Never or rarely =1
- Sometimes = 2
- Often =3
- Most of the time =4
